# A new 3D phase unwrapping method by region partitioning and local polynomial modeling in abdominal quantitative susceptibility mapping

**DOI:** 10.3389/fnins.2023.1287788

**Published:** 2023-11-15

**Authors:** Junying Cheng, Manli Song, Zhongbiao Xu, Qian Zheng, Li Zhu, Wufan Chen, Yanqiu Feng, Jianfeng Bao, Jingliang Cheng

**Affiliations:** ^1^Department of Magnetic Resonance Imaging, The First Affiliated Hospital of Zhengzhou University, Zhengzhou, China; ^2^Department of Radiotherapy, Cancer Center, Guangdong Provincial People’s Hospital, Guangdong Academy of Medical Science, Guangzhou, China; ^3^College of Software Engineering, Zhengzhou University of Light Industry, Zhengzhou, China; ^4^School of Biomedical Engineering and Guangdong Provincial Key Laboratory of Medical Image Processing, Southern Medical University, Guangzhou, China

**Keywords:** magnetic resonance imaging, phase unwrapping, region partitioning, local polynomial model, abdominal quantitative susceptibility mapping

## Abstract

**Background:**

Accurate phase unwrapping is a critical prerequisite for successful applications in phase-related MRI, including quantitative susceptibility mapping (QSM) and susceptibility weighted imaging. However, many existing 3D phase unwrapping algorithms face challenges in the presence of severe noise, rapidly changing phase, and open-end cutline.

**Methods:**

In this study, we introduce a novel 3D phase unwrapping approach utilizing region partitioning and a local polynomial model. Initially, the method leverages phase partitioning to create initial regions. Noisy voxels connecting areas within these regions are excluded and grouped into residual voxels. The connected regions within the region of interest are then reidentified and categorized into blocks and residual voxels based on voxel count thresholds. Subsequently, the method sequentially performs inter-block and residual voxel phase unwrapping using the local polynomial model. The proposed method was evaluated on simulation and *in vivo* abdominal QSM data, and was compared with the classical Region-growing, Laplacian_based, Graph-cut, and PRELUDE methods.

**Results:**

Simulation experiments, conducted under different signal-to-noise ratios and phase change levels, consistently demonstrate that the proposed method achieves accurate unwrapping results, with mean error ratios not exceeding 0.01%. In contrast, the error ratios of Region-growing (N/A, 84.47%), Laplacian_based (20.65%, N/A), Graph-cut (2.26%, 20.71%), and PRELUDE (4.28%, 10.33%) methods are all substantially higher than those of the proposed method. *In vivo* abdominal QSM experiments further confirm the effectiveness of the proposed method in unwrapping phase data and successfully reconstructing susceptibility maps, even in scenarios with significant noise, rapidly changing phase, and open-end cutline in a large field of view.

**Conclusion:**

The proposed method demonstrates robust and accurate phase unwrapping capabilities, positioning it as a promising option for abdominal QSM applications.

## 1. Introduction

The acquired complex signal in magnetic resonance imaging (MRI) is composed of both magnitude and phase components. While the magnitude is typically used, the phase is often disregarded in clinical diagnosis (Chavez et al., 2002). However, accurately recovering the underlying true phase is crucial for various clinical applications, such as water-fat separation (Ma, 2008), susceptibility weighted imaging (Rauscher et al., 2005; Sehgal et al., 2005), human brain phase imaging (Liu et al., 2011; Robinson et al., 2017), and quantitative susceptibility mapping (Wang and Liu, 2015; Langkammer et al., 2018; Lu et al., 2018; Jerban et al., 2019; Zhang et al., 2019; Yan et al., 2021; Jang et al., 2022; Chen et al., 2023). The phase is typically calculated using the arctangent function and falls within the range of (−π, π) radians (Cusack and Papadakis, 2002; Jang et al., 2019). If the underlying true phase value falls outside this range, it introduces a phase wrap. These wrapped phases are corrected by adding multiples of 2π to the phase values of neighboring voxels where the phase difference exceeds π. This correction is made under the assumption that the true phase is smooth (Dymerska et al., 2021). However, obtaining the underlying true phase is not always straightforward, particularly in cases where the signal-to-noise ratio (SNR) is low, and the phase difference between neighboring voxels in the region of interest (ROI) exceeds π.

Many phase unwrapping methods (Ghiglia and Pritt, 1998; Strand et al., 1999; Langley and Zhao, 2009; Arevalillo-Herráez et al., 2016; Robinson et al., 2017) have been proposed to tackle this challenging issue, and a comprehensive review can be found in reference (Robinson et al., 2017). These methods can be broadly classified into path-following techniques (Ghiglia and Pritt, 1998; Chavez et al., 2002; Cusack and Papadakis, 2002; Arevalillo-Herráez et al., 2016) and global optimization algorithms (Friedlander and Francos, 1996; Ghiglia and Pritt, 1998; Strand et al., 1999; Langley and Zhao, 2009). Path-following methods unwrap phase data by detecting 2π phase discontinuities between adjacent pixels along a predetermined path. While path-following approaches are efficient, they are susceptible to unwrapping errors in regions with low SNR of the phase data. These errors can propagate and accumulate, leading to residual wraps in regions with high SNR (Cheng et al., 2018a,b). Global optimization methods, on the other hand, use mathematical functions to model the underlying true phase data and perform optimization calculations to eliminate phase aliasing. While these methods are robust to noise and accurate, finding a global optimization solution is computationally expensive (Dong et al., 2017; Dymerska et al., 2021).

**FIGURE 1:**
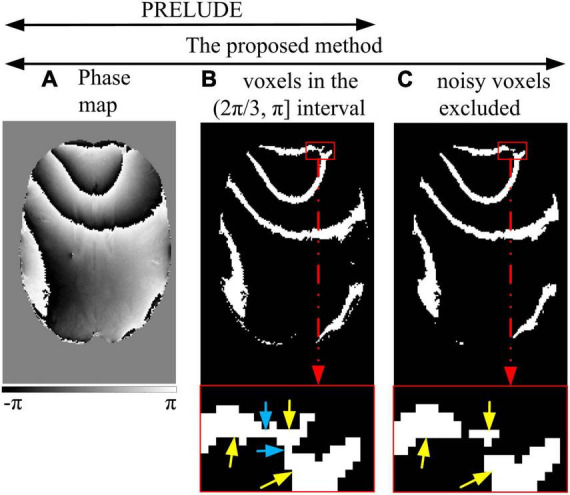
Noisy voxels excluded. PRELUDE: the voxels with phase values **(A)** within one of the defined intervals are identified **(B)**. The region in the red enlarged window **(B)** contains three areas (yellow arrows) connected by the noisy voxels (blue arrow). The phase difference between the areas is more than 2π, and hidden wraps exist. The proposed method: the first step is the same as PRELUDE **(B)**. Then, the noisy voxels are excluded, and the connected components are reidentified as initial regions **(C)**.

Region split-merging methods were developed to reconcile path-following approaches and global optimization algorithms (Zhou et al., 2021). One notable representative is the phase region expanding labeler for unwrapping discrete estimates (PRELUDE) (Jenkinson, 2003), which is considered a gold standard due to its high accuracy and widespread use in MR phase images (Karsa and Shmueli, 2018). PRELUDE employs a phase partition approach, segmenting the phase interval (−π, π) into evenly spaced subintervals, to divide the phase volume into segments of a specific value range (see Figures 1A, B). It is assumed that these regions do not contain intra-region wraps. These regions are matched and merged in a Region-growing path, assuming spatial smoothness of the phase. The unwrapping process begins with the pair of neighboring regions with the largest number of interfacing voxels on the border. However, if the initial region contains areas with a phase difference exceeding 2π (see Figure 1B), it indicates a wrap, and PRELUDE fails (Cheng et al., 2018b; Karsa and Shmueli, 2018). This situation typically arises in regions with low SNR and/or rapid phase variation in high-resolution imaging.

In this study, we introduce a novel 3D split-merging phase unwrapping method based on region partitioning and a local polynomial model. The proposed method initially applies the phase partitioning technique used in the PRELUDE approach to generate the initial regions. Noisy voxels connected to areas in the initial regions are eliminated and clustered into residual voxels. The connected regions in the ROI are reidentified and clustered into blocks and residual voxels by thresholding the number of voxels in regions. Subsequently, the proposed method sequentially performs inter-block and residual voxel phase unwrapping using the local polynomial model. We evaluate the performance of the proposed method through 3D simulations and *in vivo* abdominal data, comparing it with Region-growing (Weill Cornell Medicine, 2020), Laplacian_based (Schofield and Zhu, 2003), Graph-cut (Dong et al., 2017) and PRELUDE (Jenkinson, 2003) methods.

## 2. Materials and methods

### 2.1. The problem of phase unwrapping

The relationship between the calculated (wrapped) phase φ and the smooth true phase (unwrapped) φ, determined by the arctangent function, is defined by multiples of 2π, i.e.,


(1) $$\varphi_{\left(x,y,z\right)}=\phi_{\left(x,y,z\right)}-2\,k_{\left(x,y,z\right)}\,\pi ,$$

where (*x*, *y*, *z*) represents the spatial index of a voxel, and *k* is an integer. Phase unwrapping is achieved by adding the correct phase offset 2*k*π to the wrapped phase of each voxel, thereby recovering the underlying true phase. This is done under the assumption that the true phase exhibits spatial smoothness (Ghiglia and Pritt, 1998).

### 2.2. Related work: modeling true phase using local polynomial function

Mathematical functions are commonly employed to model the underlying true phase data, transforming the unwrapping problem into a parameter estimation problem in many phase unwrapping methods (Friedlander and Francos, 1996; Liu W. et al., 2013; Cheng et al., 2018b). However, directly modeling the true phase across a large field of view proves challenging and inefficient. In this study, a polynomial function, known for its robustness to noise and ability to estimate the true phase in two disconnected areas within the region of interest (ROI) (Cheng et al., 2018b), is used to model the underlying true phase in a local window. This function is expressed as follows:


(2) $$r_{\left(x,y,z\right)}=\phi_{\left(x,y,z\right)}-\sum_{l=0}^{L}\sum_{m=0}^{M}\sum_{n=0}^{N}C_{l,m,n}\,x^{l}\,y^{m}\,z^{n},$$

where *r*_*(x,y,z)*_ represents the fitting error; *C*_*l,m,n*_ denotes the fitting coefficients; and *L*, *M*, and *N* are the orders of the function in the *x*, *y*, and *z* directions, respectively. The least square method can be used to determine the fitting coefficients *C*_*l,m,n*_ by exploiting the information of the unwrapped voxels in the local window (Cheng et al., 2018b). The integer compensation *k* of the growing voxel (*x*_0_, *y*_0_, *z*_0_) is calculated as follows:


(3) $$k_{\left(x_{0},y_{0},z_{0}\right)}=r\,o\,u\,n\,d\,\left(\frac{X_{\left(x_{0},y_{0},z_{0}\right)}\,\hat{c}-\varphi_{\left(x_{0},y_{0},z_{0}\right)}}{2\,\pi }\right),$$

where $X_{\left(x_{0},y_{0},z_{0}\right)}=\left[1,x_{0},y_{0},z_{0},x_{0}\,y_{0}\,z_{0},\ldots ,x_{0}^{L},y_{0}^{M},z_{0}^{N},x_{0}^{L}\,y_{0}^{M}\,z_{0}^{N}\right]$ is the polynomial basis of the growing voxel (*x*_0_, *y*_0_, *z*_0_), and round(*z*) is a function that computes the closest integer to *z*.

### 2.3. Region partitioning

Figure 1 illustrates the process of excluding noisy voxels. Similar to the PRELUDE method, the phase interval of (−π, π) is divided into six evenly spaced subintervals [(−π, −2π/3), (−2π/3, -π/3), (−π/3, 0), (0, π/3), (π/3, 2π/3), and (2π/3, π)] (Figure 1), providing the fewest wrap-free initial regions (Karsa and Shmueli, 2018). For each subinterval, a mask is generated to identify the voxels whose phase values fall within that subinterval. The initial regions are identified by detecting the connected components (Figure 1B). It is worth noting that the initial regions may be comprised of areas (indicated by yellow arrows in Figure 1B) connected by a few noisy voxels, where the phase difference between these areas exceeds 2π. Consequently, the phase in the initial regions may contain a hidden wrap (indicated by the blue arrow in Figure 1B).

To prevent the initial regions from containing hidden wraps, the noisy voxels connected to the areas in the initial regions are excluded before the connected 3D regions are determined (Figure 1C). The excluded voxels meet the following criteria: (i) they are located at the edge of the identified block, and (ii) they have zero-valued first, second, or third neighbors in at least two of the three (*x*, *y*, and *z*) directions. These excluded voxels are classified as the first residual voxels (Figure 1C). Note that edge voxels inside the imaging object will be classified as the first residual voxels, so the effect on the phase unwrapping for other voxels is negligible. The 3D connected regions are reidentified. To mitigate the impact of small regions, the reidentified regions within the ROI are further classified into blocks and the second residual voxels based on a threshold applied to the voxel count of each region.

### 2.4. Regions unwrapping and merging

The blocks are initially matched and merged using the local polynomial function defined in equation 2. The block with the highest voxel count is selected as the starting block, and the phase inside it is unwrapped. The block nearest to the already unwrapped regions is chosen as the block to be unwrapped, based on the Euclidean distance between the closest voxels. The local polynomial model method is applied to the voxels in the growing block that are closest to the unwrapped regions, as well as to the voxels in the unwrapped regions that are closest to the growing block. This process yields the optimal integer offset for the growing block.

Once all blocks are matched and merged, the residual voxels are unwrapped using the quality-guided Region-growing local polynomial method. The phase derivative (Cheng et al., 2023) is calculated as the quality criterion to enhance the unwrapping of the residual voxels. During the residual unwrapping process, the second residual voxels are unwrapped first.

## 3. Experiments

### 3.1. Simulations

**FIGURE 2:**
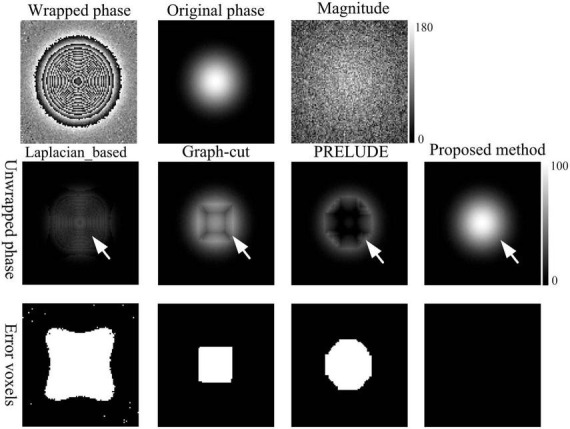
Phase-unwrapping results of the first simulation under different SNRs by using the Laplacian_based, Graph-cut, PRELUDE, and proposed methods. Due to its overall poor performance, the results for Region-growing have not been shown. The white arrows in the second row points to where obvious residual wraps in the unwrapped results. The images in the third row display the incorrectly unwrapped voxels. Voxels were considered incorrectly unwrapped if the absolute phase difference between the unwrapped phase and reference phase exceeded π/10 radians. The reference phase image was defined as the sum of the generated original phase and the phase changes caused by noise.

To assess the performance of the proposed method across varying signal-to-noise ratios (SNRs), a synthetic dataset of dimensions 100 × 100 × 100 was generated using a Gaussian function with a standard deviation (SD) of 20 voxels (Cusack and Papadakis, 2002; Cheng et al., 2018b). The magnitude of the simulation data ranged from 10 to 100 in increments of 10. Gaussian noise with a mean of zero and SD of 20 was added to the original synthesized complex data. Consequently, the SNRs of the simulated data varied from 0.5 to 5 in increments of 0.5 (Cheng et al., 2018a), as depicted in Figure 2.

A more comprehensive and complex simulation, sized at 101 × 101 × 51, was created to evaluate the proposed method under different levels of phase variation (Abdul-Rahman et al., 2016). The phase values were obtained as


(4) $$\phi_{\left(x,y,z\right)}=H\,e\,i\,g\,h\,t\times \left[\left(\frac{\sin\left(x\right)}{\pi }\right)\,\left(1.50-z\right)+\left(\frac{\sin\left(y\right)}{\pi }\right)\,\left(0.49+z\right)\right],$$

**FIGURE 3:**
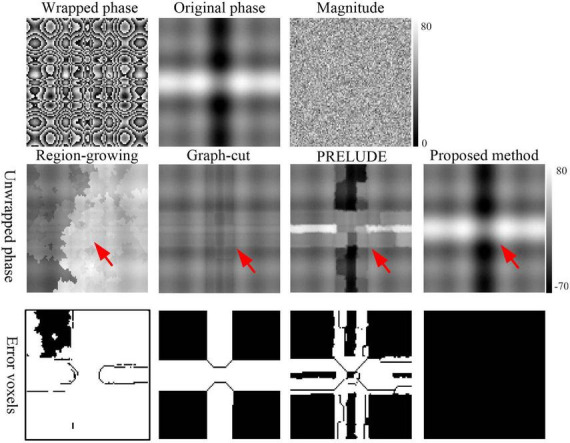
Phase-unwrapping results of the second simulation under different phase change levels along *z*-axis direction by using the Region-growing, Graph-cut, PRELUDE, and proposed methods. Due to its overall poor performance, the results for Laplacian_based method have not been shown. The red arrows in the second row points to where obvious residual wraps in the unwrapped results. The images in the third row display the incorrectly unwrapped voxels. Voxels were considered incorrectly unwrapped if the absolute phase difference between the unwrapped phase and reference phase exceeded π/10 radians. The reference phase image was defined as the sum of the generated original phase and the phase changes caused by noise.

The Height was set at 5. The magnitude was set at 50. Gaussian noise with a mean of zero and SD of 10 was added to the original synthesized complex data. The phase change levels varied along the *z*-axis direction. The resulting SNR of the generated simulation was 5, as illustrated in Figure 3.

### 3.2. *In vivo* abdominal QSM data

To validate the effectiveness of the proposed method on *in vivo* data, abdominal quantitative susceptibility mapping (QSM) data were acquired from five adult volunteers using a 3.0T MR scanner (Siemens Prisma; Siemens, Erlangen, Germany) (Bechler et al., 2019). A 2D gradient echo breath-hold sequence was employed to obtain the original data for testing and comparison of the phase unwrapping methods. The scanning parameters for the abdominal QSM data were: repetition time (TR) = 71 ms, echo time (TE) = 4.92 ms, matrix size = 256 × 256 × 10, flip angle (FA) = 20°, resolution = 1.95 mm^3^ × 1.95 mm^3^ × 5 mm^3^, bandwidth = 930 Hz/pixel, accelerated factor = 2, and scan time = 7.6 s.

The study was conducted in accordance with the Declaration of Helsinki (as revised in 2013), approved by the Ethics Board of the First Affiliated Hospital of Zhengzhou University (No. 2018-KY-88), and informed consent was obtained from all participants.

### 3.3. Implementation and parameters

The proposed method was implemented using MATLAB (R2021b; MathWorks, Natick, MA, USA) on a desk computer (Dell, Intel^®^ Core™ i7-11700, 32 GB RAM). The classical Region-growing, Laplacian_based, and Graph-cut methods, implemented in the QSM toolkit (Weill Cornell Medicine, 2020), and PRELUDE with default parameters from the fMRI software library (FSL; Oxford Center for Functional Magnetic Resonance Imaging of the Brain, UK) (Smith et al., 2004), were used for comparison with the proposed method. A mask was employed in all five methods to enhance computational efficiency. The edge detection approach outlined in reference (Cheng et al., 2018b) was utilized to generate the mask.

The parameters of the proposed method included a 26-connected neighborhood, allowing for consideration of phase changes in all directions, which was used to exclude noisy voxels in the initial regions. To mitigate the impact of small regions, typically containing phases with rapid variation and/or low SNR, regions with fewer than 100 voxels were classified as second residual voxels. The polynomial orders *L*, *M*, and *N* were all set to 2, in line with the references (Cheng et al., 2018a). In inter-block unwrapping, 100 voxels closest to the unwrapped regions in the growing block, and the 100 voxels nearest to the growing block in the unwrapped regions, were selected as fitting points. The fitting size of 100 voxels was experientially determined to be equal to the minimum block size, consistent with the reports (Cheng et al., 2018a,b). The fitting window size was set to 11, based on observations from simulation results under different SNRs. The parameters mentioned above are consistent in the simulation and *in vivo* experiments.

To quantitatively and qualitatively assess the performance of the proposed method, the misclassification ratio (MCR) (Jenkinson, 2003) was computed. The MCR represented the percentage of incorrectly unwrapped voxels in the region of interest (ROI). Voxels were considered incorrectly unwrapped if the absolute phase difference between the unwrapped phase and reference phase exceeded π/10 radians. The reference phase image was defined as the sum of the generated original phase and the phase changes caused by noise (Liu W. et al., 2013). The simulation was repeated 50 times, and the corresponding means and SDs of MCRs were calculated.

To evaluate the performance of the proposed method on *in vivo* abdominal QSM data, acquired multi-echo gradient echo (GRE) data were used for QSM reconstruction. First, a nonlinear least square fitting method (Liu T. et al., 2013) was applied to obtain the wrapped total field map from the multi-echo magnitude and phase data. Subsequently, the Region-growing, Laplacian_based, Graph-cut, PRELUDE, and proposed methods were used to obtain the unwrapped total field map. Variable-radius Sophisticated Harmonic Artifact Reduction for Phase Data (V-SHARP) method (Fang et al., 2017) was then utilized to exclude background fields, obtaining the tissue field. Finally, the STAR-QSM algorithm (Fang et al., 2017) was employed for the inversion of the tissue field into QSM maps. V-SHARP and STAR-QSM, with default parameters (Li et al., 2014), were used. If incorrectly unwrapped voxels were present in the ROI obtained by the five phase-unwrapping methods, the generated susceptibility results would likely contain serious artifacts.

## 4. Results

### 4.1. Evaluation of simulation data

Figure 2 displays representative unwrapped results of simulated data with varying SNRs using the Laplacian_based, Graph-cut, PRELUDE, and proposed methods. Images generated by Laplacian_based, Graph-cut, and PRELUDE methods exhibit noticeable residual wraps (indicated by white arrows), whereas the proposed method yields a smooth phase. Figure 3 presents representative unwrapped results of simulated data with different phase change levels along the *z*-axis direction using the Region-growing, Graph-cut, PRELUDE, and proposed methods. The Region-growing approach yields the least favorable result, while results from Graph-cut and PRELUDE are suboptimal. In contrast, the proposed method acquires perfectly unwrapped phase data.

Table 1 provides the means and standard deviations (SDs) of the misclassification ratio (MCR) for the five methods on the two simulated datasets. For the first simulated dataset, the means and SDs of the MCRs are as follows: Laplacian_based (20.65 ± 0.16), Graph-cut (2.26 ± 0.02), PRELUDE (4.28 ± 0.06), and proposed method (0.01 ± 0.01). In the case of the second simulated dataset, the means and SDs of the MCRs are as follows: Region-growing (84.47 ± 6.62), Graph-cut (20.71 ± 1.19), PRELUDE (10.33 ± 9.80), and proposed method (0.01 ± 0.01). Notably, the proposed method yields the most accurate results across both simulated datasets.

**TABLE 1:** The means and SDs of the misclassification ratio (MCR) for the Region-growing, Laplacian_based, Graph-cut, PRELUDE and proposed methods on the two simulated over 50 repetitions.

Simulation	Region-growing	Laplacian_based	Graph-cut	PRELUDE	Proposed method
1st	N/A	20.65 ± 0.16	2.26 ± 0.02	4.28 ± 0.06	0.01 ± 0.01
2nd	84.47 ± 6.62	N/A	20.71 ± 1.19	10.33 ± 9.80	0.01 ± 0.01

Values are given in means ± standard deviations; MCR, the percentage of incorrect unwrapping voxels in the region of interest.

### 4.2. Performance on *in vivo* abdominal QSM data

**FIGURE 4:**
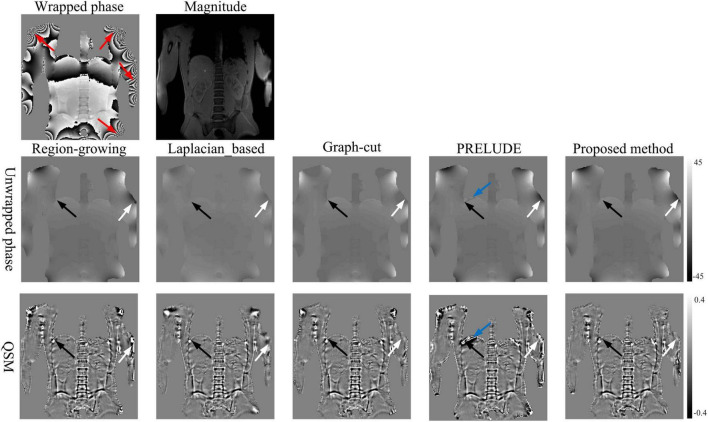
The phase-unwrapping and QSM results of the *in vivo* abdominal data by using the Region-growing, Laplacian_based, Graph-cut, PRELUDE, and proposed methods. Images in the first row are phase and magnitude. Images in the second row are the unwrapped phase by using the five methods. Images in the third row are susceptibility results from the phase data after unwrapping with every algorithm, background field removal, and dipole inversion. Red arrows indicate the position where the open-end cutlines locate at. White arrows indicate errors in susceptibility from previous discontinuities in phase unwrapping. Black arrows indicate streaking artifacts resulting from the significantly lower susceptibility of the nearby ribs. Blue arrows in the second and third rows indicate the obvious residual wraps and susceptibility artifacts. That is because the initial region in PRELUDE method contains the areas with phase difference larger than 2π.

Figure 4 illustrates the phase-unwrapping and quantitative susceptibility mapping (QSM) results of abdominal data using Region-growing, Laplacian_based, Graph-cut, PRELUDE, and proposed methods. The open-end cutlines (Chavez et al., 2002) at the edge of the wrapped phase map (indicated by red arrows in the first row) lead to phase discontinuities (indicated by white arrows in the second row) in results obtained by all five methods. However, the proposed method displays the unwrapped phase with the fewest wrapping residues. The resulting susceptibility maps from the five algorithms exhibit errors due to residual wraps (indicated by white arrows in the third row), whereas the proposed method displays susceptibility results with the least artifacts. Artifact was labeled with the assistance of a technician with 5-year experience, and was compared to the similar abdominal QSM images in the reference (Bechler et al., 2019). The results by PRELUDE contain obvious residual wraps and susceptibility artifacts (indicated by blue arrows in the second and third rows). That is because the initial region in PRELUDE method contains the areas with phase difference larger than 2π. All five algorithms show streaking artifacts near the ribs (indicated by black arrows in the third rows), which may be attributed to the significantly lower susceptibility of the ribs (Bechler et al., 2019). Overall, the proposed algorithm provides a susceptibility map with the fewest artifacts. The unwrapped and QSM results on *in vivo* brain QSM data generated by the Graph-cut, PRELUDE, and proposed methods were reported in the Supplementary Data Sheet 1.

## 5. Discussion

In this study, we have introduced a novel, robust 3D phase unwrapping algorithm based on region partitioning and a local polynomial model, aiming to enhance the state-of-the-art method, PRELUDE. The proposed method initiates with the phase partitioning approach to generate initial regions similar to PRELUDE. Noisy voxels connected to the areas in the initial regions were excluded and clustered into residual voxels. The connected regions in the region of interest (ROI) were reidentified and categorized into blocks and residual voxels based on a voxel count threshold. Subsequently, the proposed method performs inter-block and residual voxel phase unwrapping sequentially using the local polynomial model. The simulation and *in vivo* abdominal QSM experiments demonstrate that the proposed method yields unwrapped results with minimal residual wraps, significantly reducing artifacts in the abdominal QSM images.

The performance of phase unwrapping methods is generally influenced by two key factors: the sequence in which voxels are unwrapped, and the model used to estimate the underlying true phase (Ghiglia and Pritt, 1998). The proposed method categorizes voxels within the region of interest (ROI) into easily unwrappable blocks and more challenging residual voxels. The blocks are initially matched and merged, followed by unwrapping of the residual voxels using information from already corrected regions. To prevent voxels with phase differences larger than 2π in each initial region, noisy voxels connected to the areas in the initial regions, generated by the phase partition method, are eliminated and unwrapped at the end. This voxel classification strategy helps to prevent problematic voxels from emerging early in the unwrapping sequence, thus reducing the likelihood of error propagation and accumulation, as discussed in reference. The local polynomial model, which is robust against noise, is used to estimate the smooth phase in the proposed method, replacing the cost function used in PRELUDE (Karsa and Shmueli, 2018). As a result, the proposed method can accurately unwrap phase data even when adjacent voxels exhibit phase differences larger than π.

The performance of the proposed method hinges on the selection of the clustering threshold for the initial regions. A large threshold can decrease the number of blocks and increase distances between them, potentially compromising the effectiveness of modeling inter-block phase variation with the local polynomial function. Small blocks often contain voxels with significant noise or rapidly changing phase. A small threshold may not effectively mitigate the impact of small blocks, which may emerge early in the growing path and lead to results with residual wraps. In this study, the threshold for region classification was set empirically at 100, though not optimally. However, in both the simulated and *in vivo* abdominal QSM experiments presented here, the proposed method consistently yielded the best results using this threshold. In future work, it will be important to further investigate the optimization of the region classification threshold.

The parameters in the local polynomial model play a crucial role in the proposed method. In this study, the underlying true phase in a window is estimated using a 2nd-order polynomial model. The influence of polynomial order has been discussed in the reference (Cheng et al., 2023), there is no need to elaborate further. Even in the presence of rapid phase variation and significant noise, the proposed method produced satisfactory results, consistent with previous reports (Friedlander and Francos, 1996; Liang, 1996). It is imperative to carefully select the fitting data in the phase unwrapping process. Proper selection of fitting window size in equation 2 is very important to obtain the fitting data. A large window size can obtain more fitting data that may improve the robustness, but will reduce the fitting accuracy because the polynomial function may not accurately model the phase variation (Friedlander and Francos, 1996; Liang, 1996; Cheng et al., 2023). To accurately model the underlying true phase during inter-block and residual voxel unwrapping steps, two different strategies were employed to determine the voxels to be fitted. For inter-block unwrapping, we selected a specific number of the closest voxels between the already unwrapped regions and the growing block. For residual voxel unwrapping, we chose a number of already unwrapped voxels around the growing voxels in a window as the fitting data. The parameters of the polynomial function were determined empirically in this study and should be meticulously fine-tuned in practical applications.

In presence of serious noise and rapid phase change, the traditional phase unwrapping methods are computationally expensive. Deep learning (Spoorthi et al., 2018; Zhou et al., 2021; Jung et al., 2022) has the potential to drastically accelerate unwrapping and generate a correct result. Whereas, deep learning cannot be directly applicated to unwrap the phase, because the location of phase wraps is not fixed for the diverse content of medical images. In addition, deep learning method require the state-of-the-art graphic processing unit (GPU) architecture and big training data, and thus is expensive. A 2D gradient echo breath-hold sequence was used to acquire *in vivo* abdominal QSM data in this study. Long acquired time will increase the difficulty to hold the breath for volunteers. The 3D ultrashort echo time quantitative susceptibility mapping (UTE-QSM) technique in references (Lu et al., 2018; Jang et al., 2019; Jerban et al., 2019) may be used to improve the data acquisition mode.

In conclusion, in this study, a novel and robust 3D phase unwrapping method is introduced and applied to abdominal quantitative susceptibility mapping (QSM) for susceptibility result generation. The proposed method initially employs the phase partition approach to generate initial regions. Noisy voxels connected to the areas in the initial regions are then eliminated and grouped into the residual voxel category. The connected regions within the ROI are reidentified and further categorized into blocks and residual voxels based on a voxel count threshold. Subsequently, the proposed method performs inter-block and residual voxel phase unwrapping sequentially using the local polynomial model. When compared to the classical Region-growing, Laplacian_based, Graph-cut, and PRELUDE methods, simulated experiments demonstrate that the proposed method consistently yields perfect unwrapped results, even in regions with low signal-to-noise ratio (SNR) and rapidly changing phase. Abdominal QSM, known for its challenges in dealing with large susceptibility changes and regions of low SNR, benefited significantly from the proposed method, obtaining superior results. Thus, the proposed method presents a promising option for abdominal QSM applications.

## Data availability statement

The raw data supporting the conclusions of this article will be made available by the authors, without undue reservation.

## Ethics statement

The studies involving humans were approved by the Ethics Board of the First Affiliated Hospital of Zhengzhou University (No.: 2018-KY-88) and informed consent was taken from all the patients. The studies were conducted in accordance with the local legislation and institutional requirements. The participants provided their written informed consent to participate in this study.

## Author contributions

JuC: Conceptualization, Formal analysis, Investigation, Methodology, Software, Writing – original draft, Writing – review and editing. MS: Writing – review and editing, Writing – original draft. ZX: Writing – review and editing. QZ: Writing – review and editing. LZ: Data curation, Writing – review and editing. WC: Writing – review and editing. YF: Writing – review and editing, Conceptualization. JB: Writing – review and editing. JiC: Supervision, Writing – review and editing.
