# Supplementary material for: A new 3D phase unwrapping method by region partitioning and local polynomial modeling in abdominal quantitative susceptibility mapping
Source: Front Neurosci. 2023 Nov 15;17:1287788. doi: 10.3389/fnins.2023.1287788 (PMC10684715; doi:10.3389/fnins.2023.1287788)
Supplement: Supplementary file 1 [file Data_Sheet_1.docx]

## Supplementary

This material is mainly used to discuss more about the reason why well-accepted Graph-cut method fails in the simulation experiments by using the simulation 2 (Abdul-Rahman et al., 2009).

Supporting Figure S1 shows the results in X, Y and Z plane on the simulated dataset 2 under the different phase change levels along the z-axis direction produced by the Graph-cut (Dong et al., 2017) and proposed methods. The images in the first and second rows are the wrapped phase and the original phase, respectively. The images in the third row display the voxels locate at the positions where the phase difference between adjacent voxels in 6-neighbourhood larger than π. The images in the fourth and fifth rows show the error voxels generated by the Graph-cut and proposed methods, respectively. There are obvious error voxels in the result with Height of 5 by Graph-cut, while proposed method obtains a perfect unwrapped phase image.

The error voxel maps obtained by the Graph-cut method is very similar to the phase difference larger than π maps (as pointed by the red arrows). That is because the energy minimization function used in Grap-cut method cannot obtain the optimal solution for the voxels with the phase difference larger than π. The proposed method categorizes voxels within the Region of Interest (ROI) into easily unwrappable blocks and more challenging first and second residual voxels. The blocks are first matched and merged, followed by unwrapping of the second and first residual voxels using information from already corrected regions. To prevent voxels with phase differences larger than 2π in each initial region, noisy voxels connected to the areas in the initial regions generated by the phase partition method, are classified as the first residual voxels and unwrapped at the end. This voxel classification strategy helps to prevent problematic voxels from emerging early in the unwrapping sequence, thus reducing the likelihood of error propagation and accumulation. The local polynomial function model, which is robust against noise, is used to estimate the smooth phase in the proposed method. As a result, the proposed method can accurately unwrap phase data even when adjacent voxels exhibit phase differences larger than π.


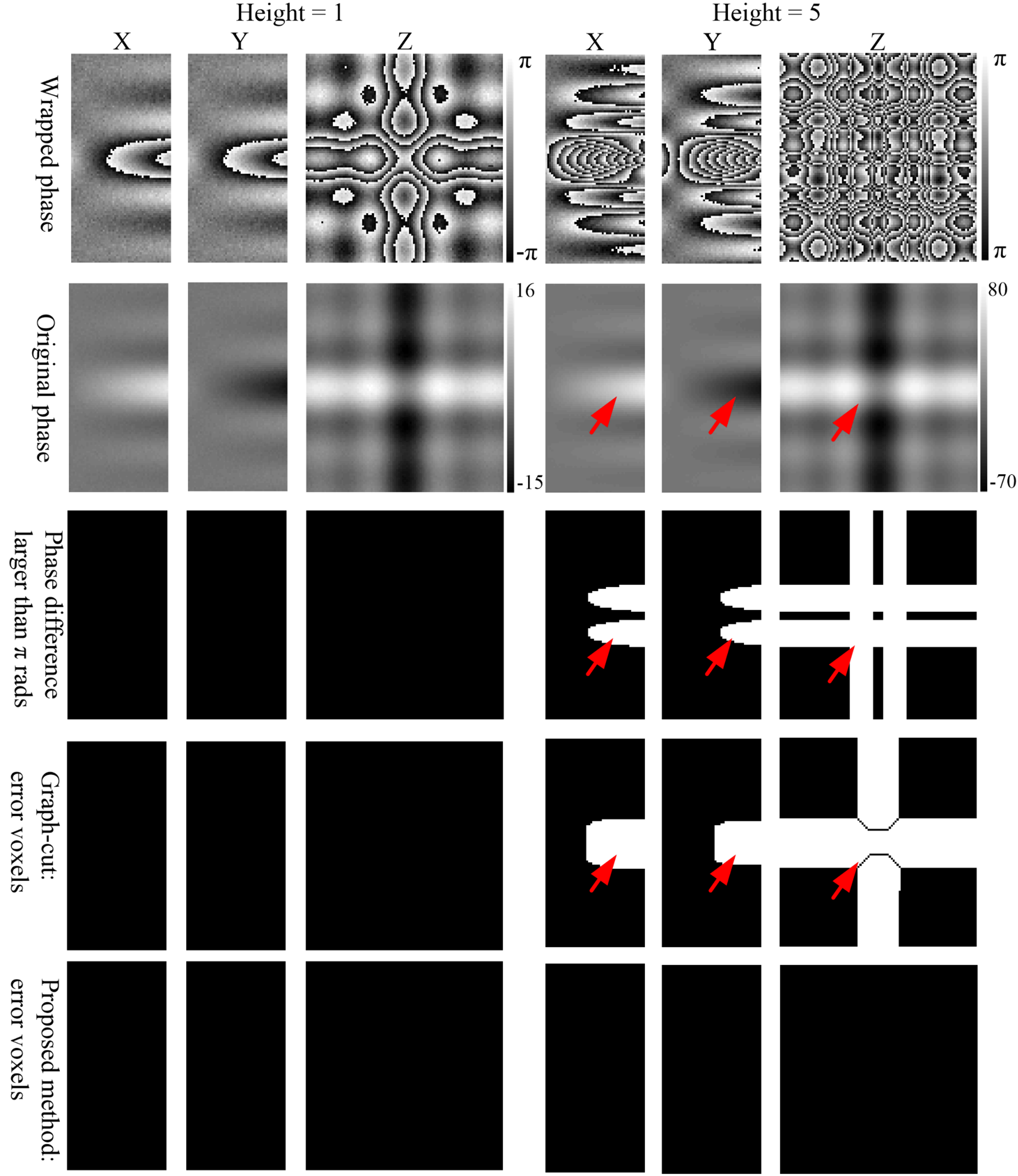


Supporting Figure S1. The results in X, Y and Z plane on the simulated dataset 2 under the different phase change levels along the z-axis direction produced by the Graph-cut and proposed methods. The error voxel maps obtained by the Graph-cut method is very similar to the phase difference larger than π images (as pointed by the red arrows). The original phase is generated by Eq. 4. Voxels were considered incorrectly unwrapped if the absolute phase difference between the unwrapped phase and reference phase exceeded π/10 radians. The reference phase image was defined as the sum of the generated original phase and the phase changes caused by noise.

To verify the validity of the proposed method on in vivo brain QSM data, we used brain QSM data collected on a 3.0T MR machine (Philips Ingenia Elition, Netherlands) from four Parkinson's disease patients. The study was conducted in accordance with the Declaration of Helsinki (as revised in 2013). The study was approved by ethics board of the First Affiliated Hospital of Zhengzhou University (No.: 2018-KY-88) and informed consent was taken from all the patients. A 3D multi-echo gradient-echo sequence was used to obtain the data for comparing the Graph-cut, PRELUDE, and proposed methods. The scanning parameters of the brain QSM data were: repetition time (TR) = 45 ms, Echo time 1 (TE1) = 7.20 ms, echo space = 7 ms, echo number = 4, matrix size = 288×288×70, flip angle (FA) = 20°, resolution = 0.76×0.76×0.80 mm^3^, bandwidth = 152.0 Hz/pixel, and scan time = 4.6 minutes.

To evaluate the performance of the Graph-cut, PRELUDE, and proposed methods on in vivo brain QSM data, the acquired in vivo 3D multi-echo GRE brain data was used to reconstruct QSM. The wrapped total field map was firstly generated by a non-linear least square fitting method. Subsequently, the unwrapped total field maps were obtained by the Graph-cut, PRELUDE and CLOSED methods. After that, the projection onto dipole fields (PDF) method (Liu T et, al. 2011) was used to exclude the background fields to obtain the tissue field. Finally, the morphology enabled dipole inversion (MEDI) algorithm (Liu J et, al. 2012) was used to inverted the tissue field to the QSM maps. The PDF with default parameters, and MEDI with λ of 1000 in the MEDI_toolbox (MEDI, 2020) were used. If the phase unwrapping method obtain the phase images with residual wraps, the QSM maps will contain susceptibility artifacts.

Supporting Figure S2 shows the unwrapped and QSM results in X, Y and Z plane on in vivo brain data produced by the Graph-cut, PRELUDE, and proposed methods. The white arrows in the first column point to where exist the subtle difference in the phase images. The white arrows in the fourth column point to where exist the subtle difference in the QSM images. The residual wraps in the phase images generated by the Graph-cut and PRELUDE methods lead to susceptibility artifacts in the corresponding QSM results. Whereas, the QSM images using the phase generated by the proposed method contain no obvious artifact, and the morphological structure is similar to the magnitude image (white arrow in the first row).


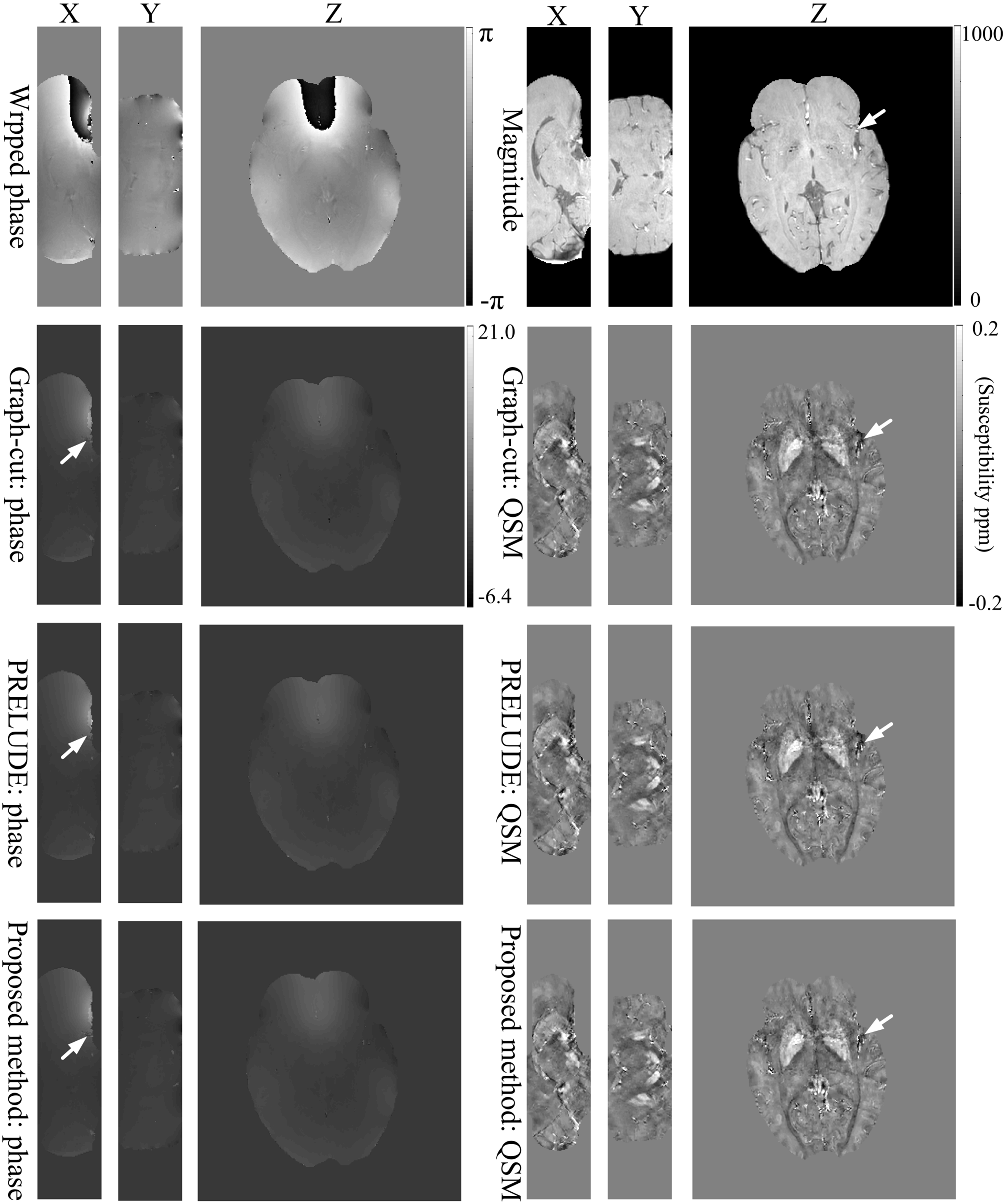


Supporting Figure S2. The unwrapped and QSM results in X, Y and Z plane on in vivo brain data produced by the Graph-cut, PRELUDE, and proposed methods. The white arrows in the first column point to where exist the subtle difference in the phase images. The white arrows in the fourth column point to where exist the subtle difference in the QSM images. The residual wraps in the phase images generated by the Graph-cut and PRELUDE methods lead to susceptibility artifacts in the corresponding QSM results.

The quantitative analysis of QSM values in deep gray matter nuclei in Parkinson's disease patients was presented. We have shown the two representative axial slices of QSM in supporting Figure S3. The subcortical structures of the targeted deep gray matter nuclei were directly identified and manually segmented on QSM images by using the ITK-SNAP software (Yushkevich et al., 2006). The mean and SDs of QSM values of the subcortical structures were reported in supporting Table S1. The variations of the QSM values in the subcortical structures are basically consistent with the reference (Xu et al., 2022).


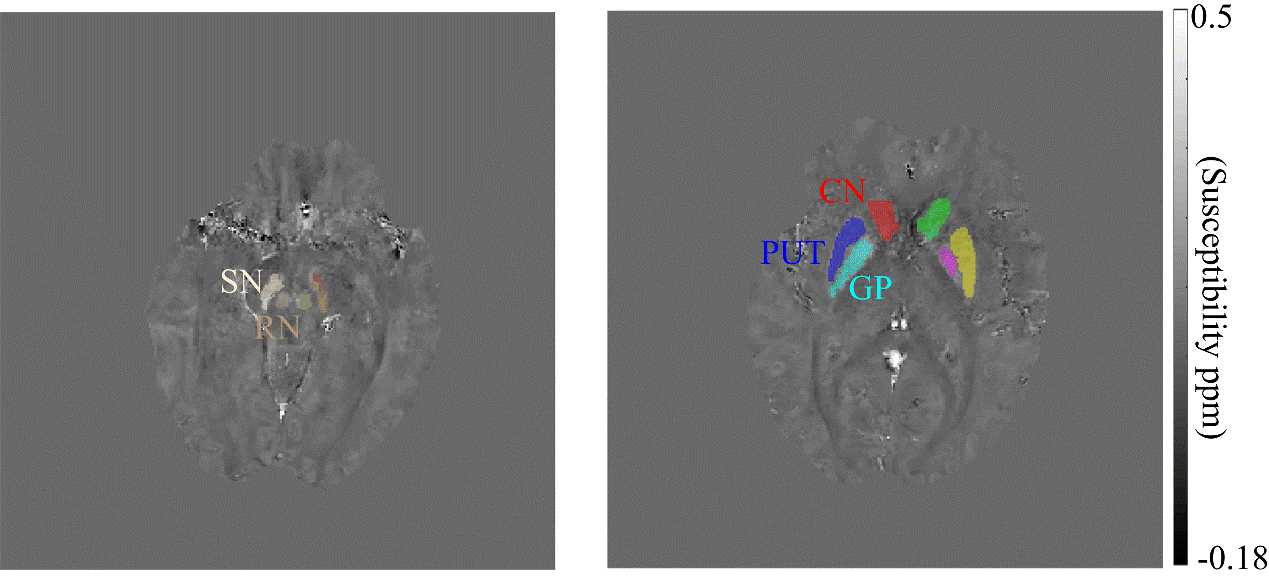


Supporting Figure S3. Two representative axial slices of QSM images from the Parkinson's disease patients with color overlay of targeted deep grey matter nuclei. SN, substantia nigra; RN, red nucleus; CN, caudate nucleus; PUT, putamen; GP, globus pallidus.

Supporting Table S1. Regional QSM values for the Parkinson's disease patients (Mean ± SDs, ppm).

| Data | Locations | SN | RN | CN | PUT | GP |
| --- | --- | --- | --- | --- | --- | --- |
| four volunteers | Left | 0.111 ± 0.021 | 0.106 ± 0.053 | 0.030 ± 0.044 | 0.048 ± 0.029 | 0.088 ± 0.037 |
|  | Right | 0.069 ± 0.025 | 0.094 ± 0.046 | 0.036 ± 0.042 | 0.054 ± 0.030 | 0.070 ± 0.031 |
|  | Both | 0.091 ± 0.022 | 0.099 ± 0.046 | 0.034 ± 0.045 | 0.053 ± 0.037 | 0.081 ± 0.035 |

To help with reading, we have added Reference here.

Weill Cornell Medicine. (2020) MEDI. http://weill.cornell.edu/mri/QSM/Online.zip. [Accessed January 30, 2020].

Abdul-Rahman, H., Arevalillo-Herráez, M., Gdeisat, M., Burton, D., Lalor, M., Lilley, F., et al. (2009). Robust three-dimensional best-path phase-unwrapping algorithm that avoids singularity loops. *Applied Optics* 48(23), 4582-4596. doi: 10.1364/ao.48.004582

Dong, J., Chen, F., Zhou, D., Liu, T., Yu, Z., and Wang, Y. (2017). Phase unwrapping with graph cuts optimization and dual decomposition acceleration for 3D high-resolution MRI data. *Magnetic Resonance in Medicine* 77(3), 1353-1358. doi: 10.1002/mrm.26174

Liu T, Khalidov I, de Rochefort L, Spincemaille P, Liu J, Tsiouris AJ, Wang Y. (2011). A novel background field removal method for MRI using projection onto dipole fields. *NMR in Biomedicine* 24(9):1129-1136. doi: 10.1002/nbm.1670

Liu J, Liu T, de Rochefort L, Ledoux J, Khalidov I, Chen W, Tsiouris AJ, Wisnieff C, Spincemaille P, Prince MR. (2012). Morphology enabled dipole inversion for quantitative susceptibility mapping using structural consistency between the magnitude image and the susceptibility map. NeuroImage 59(3):2560-2568. doi: 10.1016/j.neuroimage.2011.08.082

Jenkinson, M. (2003). Fast, automated, N-dimensional phase-unwrapping algorithm. *Magnetic Resonance in Medicine* 49(1), 193-197. doi: 10.1002/mrm.10354

Xu J, He X, Xu Y, Chen X, Li M, Zhang L, Fu X, Pan M, Wang Q, Hu X. (2022). Characteristics of systemic inflammation and brain iron deposition in Parkinson's disease patients. *ANNALS of Clinical and Translational Neurology* 9(3):276-285. doi: 10.1002/acn3.51512

Yushkevich PA, Piven J, Hazlett HC, Smith RG, Ho S, Gee JC, Gerig G. (2006). User-guided 3D active contour segmentation of anatomical structures: significantly improved efficiency and reliability. *NeuroImage* 1;31(3):1116-28. doi: 10.1016/j.neuroimage.2006.01.015
